# Arrogant or caring? Influence of transactive communication in interprofessional learning on knowledge gains and stereotype changes

**DOI:** 10.3205/zma001462

**Published:** 2021-03-15

**Authors:** Mira Mette, Martin Hänze

**Affiliations:** 1 Medical Faculty Mannheim, Heidelberg University, Division for Study and Teaching Development, Mannheim, Germany; 2 University of Kassel, Educational Psychology, Department of Psychology, Kassel, Germany

**Keywords:** interprofessional learning, transactive communication, peer tutoring, medical students, physiotherapy trainees

## Abstract

**Objective:** In interprofessional peer tutoring, medical students and physiotherapy trainees teach and practice examination techniques and work out profession-specific similarities and differences. In small interprofessional groups, we investigated the influence of transactive communication – alternately referring to and building on the statements made by the dialogue partner in the process of conveying information – on knowledge gains and changes in stereotypes of the other profession.

**Methods:** A total of 132 medical students and 48 physiotherapy trainees divided into 24 small interprofessional groups indicated the extent of their stereotypes of the other profession before and after the practice session, as well as their perceived increase in knowledge. They evaluated the group work and the perceived intensity of transactive communication. We used regression analyses to test the hypotheses.

**Results:** The intensity of transactive communication in the physiotherapy trainees was positively related to knowledge gains in the medical students. However, this did not apply to the knowledge gains in physiotherapy trainees. With regard to stereotype changes, the intensity of one's own transactive communication unexpectedly turned out to be a significant, albeit weak, influencing factor: The more intensive one's own transactive communication was, the more negative the stereotypes of the other profession became.

**Conclusion: **Transactive communication in interprofessional groups can improve the exchange of knowledge in peer tutoring and bring about changes in stereotypes. Measures to improve transactive communication, e.g. training sessions or specific communication exercises, could help to improve the effectiveness of interprofessional learning.

## Zusammenfassung

**Zielsetzung: **Im interprofessionellen Peer-Tutoring vermitteln und üben Medizinstudierende und Physiotherapieauszubildende wechselseitig Untersuchungstechniken und erarbeiten Gemeinsamkeiten und berufsgruppenspezifische Unterschiede. In interprofessionellen Kleingruppen wurde untersucht, welchen Einfluss transaktive Kommunikation, d.h. die wechselseitige Bezugnahme auf den Gesprächspartner bei der Informationsvermittlung, auf Wissenszuwachs und Veränderung der Stereotype über die andere Berufsgruppe hat.

**Methode: **Insgesamt 132 Medizinstudierende und 48 Physiotherapieauszubildende aus 24 gemischten Kleingruppen gaben die Ausprägung der Stereotype über die andere Berufsgruppe vor und nach der Übungseinheit sowie den empfundenen Wissenszuwachs an. Sie bewerteten die Gruppenarbeit und die dabei wahrgenommene Intensität der transaktiven Kommunikation. Die Hypothesenprüfung erfolgte über Regressionsanalysen.

**Ergebnisse: **Die Intensität der transaktiven Kommunikation der Physiotherapieauszubildenden hing mit dem Wissenszuwachs der Medizinstudierenden positiv zusammen. Dies galt jedoch nicht umgekehrt für den Wissenszuwachs der Physiotherapieauszubildenden. Bei der Veränderung der Stereotype zeigte sich überraschend die Intensität der eigenen transaktiven Kommunikation als signifikanter, wenn auch schwacher Einflussfaktor: je intensiver die eigene transaktive Kommunikation war, desto stärker zeigte sich eine Veränderung in Richtung negativer Stereotype über die andere Berufsgruppe.

**Schlussfolgerung: **Die transaktive Kommunikation in interprofessionellen Gruppen kann beim Peer-Tutoring den Wissensaustausch verbessern und Veränderungen der Stereotype bewirken. Maßnahmen zur Verbesserung der transaktiven Kommunikation, wie z. B. Trainings oder gezielte Übungen, könnten dazu beitragen, die Wirksamkeit interprofessionellen Lernens zu verbessern.

## Background

Collaborative skills are considered a key competence for today's professional life [1]. This is particularly true for interprofessional (IP) collaboration in the complex health care system, as one profession alone cannot ensure optimal patient care [2], [3], [4]. Effective communication between professions is essential to ensure patient safety [5]. Therefore, in addition to patient-centred communication, IP communication should also be raised and practised in health care education.

As required [6], [7], [8], [9], IP courses have been offered in Germany for several years now to prepare future health professionals for interprofessional collaboration. The results of the large number of (inter)national studies on IP learning are mainly positive [10], [11], [12], [13], [14], [15], [16], [17]: Educational research reports high participant satisfaction, more knowledge about the professions, better understanding and more positive attitudes towards IP learning and collaboration. Positive interaction and communication between the professions, which are absolutely essential to achieve the IP learning objectives [15], are described too. Although IP learning is considered to be effective for acquiring important basics for successful IP collaboration [18], there are few studies that prove this in regard to improved patient outcomes [9], [18], [19]. However, this lack of evidence does not mean that IP learning has no effect [18].

IP learning is characterised by heterogeneous IP groups. Cooperative forms of learning require all group members to contribute and interact. In this way, common knowledge about profession-specific expertise can be acquired through IP interactions [20]. At the same time, reciprocal relationships can also develop within the groups [21]. Cooperative learning can lead to better cognitive learning success and strengthen the social skills of the group members [22], [23], [24]. The intensity and quality of interaction between learners play an important role in this process [25], [26], [27], which depends, among other things, on group cohesion. For example, high group cohesion promotes mutual appreciation and interdependence which, in turn, can encourage mutual support between group members and have a positive impact on learning success [28].

When engaging in cognitive learning in cooperative group work, learners integrate new information into their existing knowledge base through processes of restructuring or elaboration [29]. Explaining learning content to someone [30], [31] is a very effective method of elaboration that is used in reciprocal peer tutoring. By alternately taking on the role of tutor or tutee, all group members can benefit twofold [32]: As a tutee, they acquire or enhance the learning content (acquisition of knowledge and skills); as a tutor, they review and convey learning content they are familiar with (in-depth review of the learning content, improved communication and presentation skills, better understanding of people from other backgrounds, higher satisfaction, greater self-confidence) [33]. Due to the greater reciprocity of active and reactive interactions between learners, reciprocal peer tutoring is more strongly characterised by intensive collaboration than tutoring with fixed roles [34].

In cooperative learning, the statements that build on each other are important [35]. Alongside the content, individual participation in the communication process and interaction between the group members are also relevant [36]. Interactions between learners are considered a consistent predictor of learning outcomes [31]. IP learning is about exchanging, substantiating, analysing and comparing profession-specific views, and developing shared solutions. The professions should be able to interact positively by supporting each other and avoiding competitive situations [37], [38]. This supportive form of communication is also called transactive communication (“trac”), where “transactive” describes the process of mutually referring to the learning partner’s ideas [39]. The concept of trac is based on the fact that statements explicitly refer to each other, complement each other, and are advanced further by the group members [40], [41]. The group members’ coherent interaction can lead to a shared elaboration (exchange, combination, and integration of learning content) and knowledge construction [39], [40], [41]. Clarifying and elaborating on transactive statements, in particular, are considered sophisticated because they refer, to a greater extent, to the other group member’s statements and strongly advance the learning process [40]. Learners benefit both from elaborating on the others’ statements and from the elaboration on their own statements by other group members [35]. The quality of the trac depends on the intensity of reciprocal references among the group members [42]. The learning success is related to the quality of the elaboration on the ideas discussed [43]. Successful trac is a high-quality form of communication that contributes to achieving collaboration and being helpful and productive for the discussion partners [29], [44].

A mandatory IP practice session (90 min) based on reciprocal peer tutoring is offered at the University Medical Centre Mannheim [45]. In small IP groups, medical students and physiotherapy trainees learn and practice the psychomotor skills of selected examination techniques (extremities and trunk) together by observing, instructing, practising and giving feedback (see table 1 (Tab. 1)).

The elements of cooperative learning [46] were taken into account when selecting the tasks. Special attention was paid to creating positive interdependence to promote trac within the IP groups and to support the shared knowledge acquisition. After the practical part, the IP groups reflected on professional similarities and differences, e.g. in terms of expertise, [47] and their impact on IP collaboration. In this way, a realistic intergroup differentiation should be achieved through intergroup contact, in which the professions positively differentiate themselves from each other [48]. Any negative perceptions or stereotypes about the other profession can thus be reduced [49] and at best be generalised in order to improve the relationships between the professions in the long term [50]. A controlled study has already examined the effectiveness of the IP practice session in terms of the levels of knowledge and stereotypes about the other profession [45]. Peer tutoring in the IP practice session was assessed to be a suitable learning method since the reciprocal approach allows the professions to complement each other and to meet at “eye level” and on a relatively informal basis. It was demonstrated, albeit weakly and in some cases only after a pretest had been applied, that knowledge was higher and stereotypes were more positive among the learners of the IP practice session. In order to learn more about the IP group work during the practice session, an additional study was carried out to investigate the perceived communication behaviour in the IP groups via the trac.

## Objective

This study examined the influence of trac in peer tutoring on knowledge gains and stereotype changes about the other profession. The trac was collected through self-assessing the intensity of the trac displayed. The trac was measured according to the frequency of information exchange within the IP group. A distinction was made between one’s own trac (the trac displayed by oneself) and the other trac (the trac displayed by the other profession in the IP group).

The following hypotheses were tested:

1. The more intensively the other trac is rated in the IP group, the more knowledge about the other profession is gained through conversations.
2. The intensity of one's own trac and the other trac in the IP group has an influence on the positive change in stereotypes about the other profession.

The study was approved by the Medical Ethics Committee II of the Mannheim Medical Faculty (label 2015-525N-MA).

## Methods

### Sample

The IP practice session took place three times in 2015 with a total of 196 learners (only the experimental group of the effectiveness study [45]). In addition to 132 medical students (3^rd^ year), 48 physiotherapy trainees (2^nd^ year) took part, with 21 trainees participating in the session twice due to organisational constraints, but with differences in the practical skills content and the group compositions with other medical students. A total of 24 IP groups (see table 2 (Tab. 2)) were randomly formed by combining one of the predetermined groups of usually 5 medical students with 2-4 physiotherapy trainees. A one-way ANOVA showed that there were no significant differences between the IP groups in terms of age, motivation, expected usefulness, previous experience of working with the other profession, or stereotypes (all p>.10).

#### Procedure and instruments

The paper-based survey was conducted among the learners. The students attending the module “Introduction to Clinical Medicine” (3^rd^ year) and the physiotherapy trainees (2^nd^ year) were asked in a pretest at the beginning of the module to rate the extent of their stereotypes regarding the other profession. Participation in the study was voluntary and anonymous. The posttest took place directly after the practical part of the practice session. The translation of stereotypical characteristics associated with health professionals [51] was used to measure the stereotypes. An acceptable scale (Cronbach's α=77, n=194) with the four items “good communicators”, “compassionate”, “aloof” (negative), and “arrogant” (negative) (cf. [45]) was achieved by removing items using “alpha if item deleted” [52].

**Figure 1:**
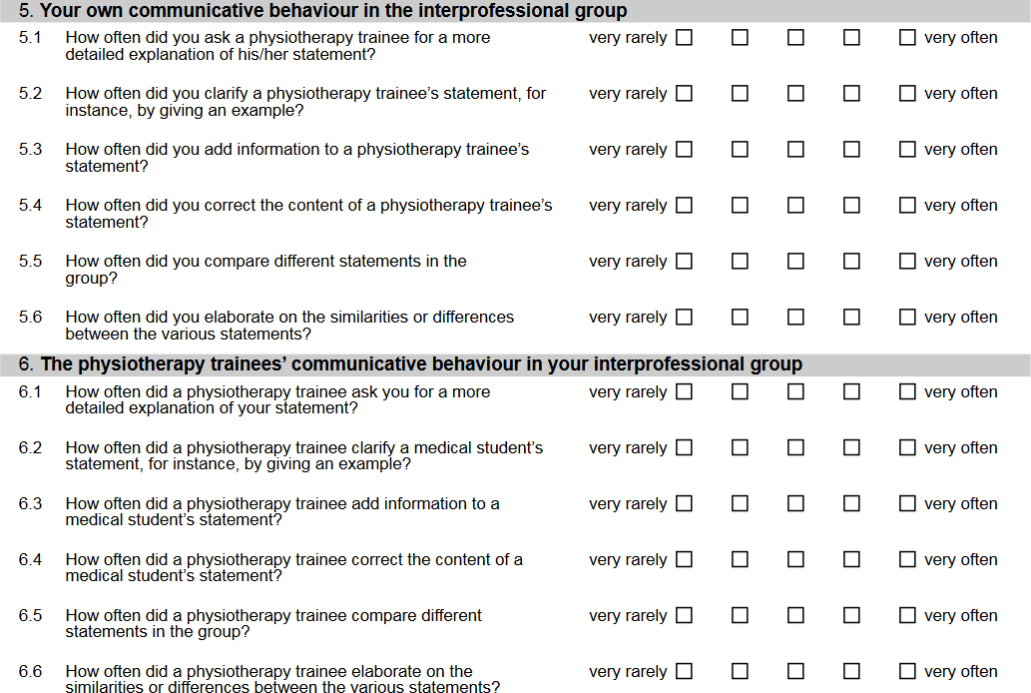
Items for measuring one's own transactive communication and the transactive communication of the other profession (medical student version).

The learners assessed the IP group work at the end of the practice session. The group climate and the trac were measured using a questionnaire to capture transactive interaction behaviour [42]. We specifically adapted the items to the actual learning context and reduced the number of items, while maintaining the breadth of content and good reliability (see figure 1 (Fig. 1)). The internal consistency and homogeneity of the scales relevant to IP group work were calculated using the data from the overall sample. In the process of removing items using “alpha if item deleted” [52], we achieved an acceptable to very good reliability and a medium to strong homogeneity [53] of the scales “one’s own trac” (α=.71, n=192, H=.40), “the other trac” (α=.80, n=189, H=.53), and “group climate” (α=.91, n=194, H=.76) by removing one item. In addition, the perceived knowledge gains about the other profession were rated. Participant satisfaction was measured using school grades (1=very good, 6=poor). The piloting of the adapted questionnaire with participants of an earlier IP practice session confirmed that the items were phrased in a clear and unambiguous way and that the different aspects of one's own trac and the other trac could be assessed well by the test persons.

#### Data analysis

We tested the hypotheses using a correlative study design with single or multiple regression analyses, separately according to profession. We analysed to what extent the variable “self-reported knowledge gains about the other profession through conversations” can be predicted by the other trac and to what extent the variable “stereotype changes about the other profession” (difference between posttest and pretest values) can be predicted by the intensity of one’s own trac and the other trac.

We used the software SPSS^®^ 22 for statistical calculations. Effect sizes were calculated.

## Results

The response rate was 96%. The practice session was rated “good” or “very good” by 78% of medical students and 85% of physiotherapy trainees. Table 3 (Tab. 3) shows significant differences between the professions for the variables knowledge gains, stereotype changes, and one’s own trac and the other trac. Compared to the physiotherapy trainees, the medical students stated that they had learned more new things through the IP conversations (small effect). The physiotherapy trainees changed their stereotypes about the other profession more than the medical students (medium effect). Both professions rated the trac of the physiotherapy trainees higher than the trac of medical students (medium effects). The control variable "group climate" could not be used due to a lack of discrimination possibilities (ceiling effect).

### Influence of the other trac on knowledge gains

A bivariate regression analysis (predictor variable: the other trac, criterion variable: knowledge gains) confirmed that the trac of physiotherapy trainees had a positive influence on the medical students’ knowledge gains about physiotherapists (F(1, 123)=26.05, p<.01, β=.43, t(123)=5.10, p<.01). However, the intensity of the trac of the medical students did not play a role in the physiotherapy trainees’ knowledge gains about doctors (F(1, 67)<1, p>.20).

#### Influence of the trac on stereotype changes

Multiple regression analyses with one’s own trac and the other trac as predictor variables and the stereotype changes as a criterion variable were carried out. It showed for all learners that the other trac did not have an influence on stereotype changes. While the regression model as a whole was not significant for the medical students (F(2, 104)=2.36, p=.10, Ra^2^=.03), the predictor variable “intensity of one’s own trac” (β=-. 24, t(104)=2.10, p<.05) was significant. The model, however, was able to explain 13% of the variance in the physiotherapy trainees (F(2, 64)=5.95, p<.01, Ra^2^=.13, β=-.46, t(64)=-3.18, p<.01). In both cases, there was a negative effect, i.e. the more intensive one's own trac was, the more negative the stereotypes about the other profession became.

## Discussion

The practice session in reciprocal peer tutoring provided the opportunity to facilitate contact and interactions between the professions in ad hoc IP groups. Even though the control variable “group climate” could not be meaningfully incorporated into the regression model due to a ceiling effect, data showed that the group climate was generally rated very positively by the learners – a good foundation for positive IP interactions. As expected, the intensity of the trac of the physiotherapy trainees had a positive effect on the medical students’ self-reported knowledge gains. The trac of the medical students was significantly perceived as less intensive by the physiotherapy trainees, and thus it could not influence their knowledge gains. This may have been due to the ratio of physiotherapy trainees to medical students. Usually medical students were in the majority in the IP groups. There could have been the impression that due to their professional knowledge the few physiotherapy trainees had communicated more intensively with the medical students than the medical students did with the physiotherapy trainees. In addition, the physiotherapy trainees were probably perceived as more proficient in explaining, demonstrating and observing practical skills, as these activities are trained from the start of the physiotherapy trainee program. As the involvement of the two professions in the reciprocal peer tutoring group work was not balanced, it was probably the physiotherapy trainees who mainly took on the tutor role and thus could benefit less from the advantages of being a tutee [33].

Regarding the influence of the trac on stereotype changes, the results were not in line with our hypothesis. Contrary to our expectation, there was a negative effect on one’s own trac, which was stronger among physiotherapy trainees. The fact that the stereotypes became more negative as the intensity of one’s own trac in the group interaction increased may be due to the fact that the portion of the other trac was reduced at the same time. A high intensity of one's own trac (e.g. by assuming the tutor role) could have led to increased ego strength in the respective tutors by perceiving their own strengths and to increased self-confidence [54]. This may have strengthened the sense of belonging to one's own profession and fostered a differentiation from the other profession. 

IP learning in the form of reciprocal peer tutoring is intended to enhance both cognitive learning and social skills through positive interactions between the professions [22], [23], [24]. Since the intensity of one's own trac and the other trac can have an influence on knowledge gains and changes in stereotypes about the other profession, learners should ideally possess transactive dialogue competence. This would enable all learners to successfully contribute to IP group work by mutually elaborating on and developing ideas, so that all learners can benefit from IP learning on a professional and social level through balanced trac.

The results on IP group work complement the statements of the quasi-experimental study [45] by shedding light on group interactions. The results on transactive communication suggest that for IP collaboration even more attention should be paid to ensuring that both professions can benefit from each other. Shared learning situations should be designed in such a way that both professions can contribute their knowledge in a similar way, thus allowing development of high-quality, mutually transactive communication. An appropriate training could further support the development of transactive dialogue competence, which, in turn, could have a positive effect on the collaboration of the dialogue partners [39].

### Limitations

The study is based on self-reported data that provide only a limited perspective on the group processes. A qualitative evaluation of the dialogue transcripts based on video/audio recordings might have added a complementary perspective. Since the participation of medical students and physiotherapy trainees in the compulsory practice session was obligatory, the data were probably not, or only slightly, distorted by a volunteer bias. The different group sizes are a problem for collecting consistent data on the other trac. For example, physiotherapy trainees could evaluate the trac of medical students by rating the trac of a single medical student, of several or of all students in their IP group. In addition, the intensity of the trac was measured solely by the frequency of certain aspects of communication. Moreover, knowledge gains about the other profession was not defined, i.e. it is not clear whether learners referred only to the subject-specific knowledge gains in the practical part, or to the results of reflection and discussion in the group, or to both. Perhaps a control variable “group climate” allowing for better differentiation or other control variables could have provided more detailed information about IP group work. We must also assume that a 90-minute session can only initiate changes in stereotypes about the other profession, but long-term effects cannot be expected. This would require regular IP contact with successful communication and cooperation.

## Conclusion

Collecting data on the perceived trac in group work made it possible to gain better insight on how IP interactions take place in reciprocal peer tutoring. A prior trac training might have increased the learners’ dialogue competence and thus might have made IP peer tutoring more effective. Educating future health professionals using the trac approach could help improve communication in general and IP communication in particular. This should be considered when developing IP courses.

## Profiles

**Name of the teaching site: **University Medical Centre Mannheim

**Field of study/profession:**

- Medical interviewing: medical studies
- Interprofessional learning: medical studies, physiotherapy training and nursing training

**Number of learners per year: **approx. 220 students/approx. 75 trainees in interprofessional learning

**Is a longitudinal communication curriculum implemented? **Yes. There is also a longitudinal interprofessional learning curriculum.

**In which terms are communication and social skills taught? **In all years of study or training programs

**Which teaching formats are used? **

- Seminars, bedside teaching with simulated patients and real patients
- The interprofessional learning curriculum including an interprofessional training ward

**In which terms are communication and social skills assessed (formative or relevant for a pass and/or graded)? **In the 3^rd^, 4^th^ and 5^th^ years of study (graded, formative)

**Which examination formats are used?**

- OSCE, graded simulation interviews
- Interprofessional training ward: interprofessional aspects are included in the evaluation and grading of the patient presentation, the progress documentation and the patient report

**Who (e.g. clinic, institution) is in charge of the development and implementation?**

- Skillslab TheSiMa in cooperation with various clinics/institutes
- Interprofessional learning curriculum: Division for Study and Teaching Development of the Medical Faculty Mannheim in cooperation with the School of Physiotherapy as well as School of Nursing of the Academy of University Medical Centre Mannheim

## Current professional roles of the authors

- Dr. phil. Mira Mette: Research assistant, Medical Faculty Mannheim of Heidelberg University, focus: interprofessional learning
- Prof. Dr. phil. Martin Hänze: Professor of Educational Psychology, University of Kassel; research areas: quality of communication in settings of cooperative learning, empirical research on teaching and learning in schools and higher education

## Competing interests

The authors declare that they have no competing interests. 

## Figures and Tables

**Table 1:**
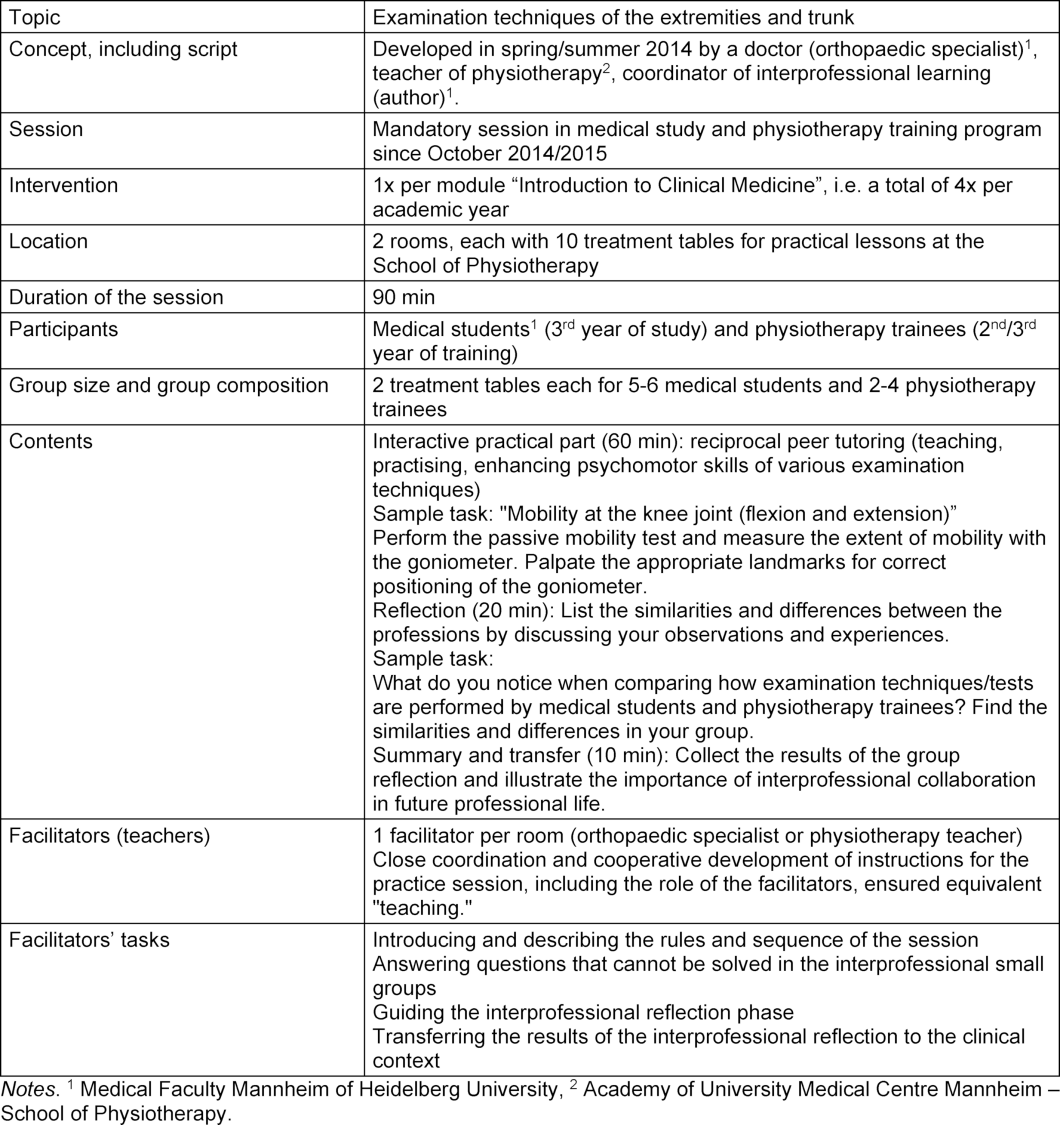
Information about the interprofessional practice session

**Table 2:**
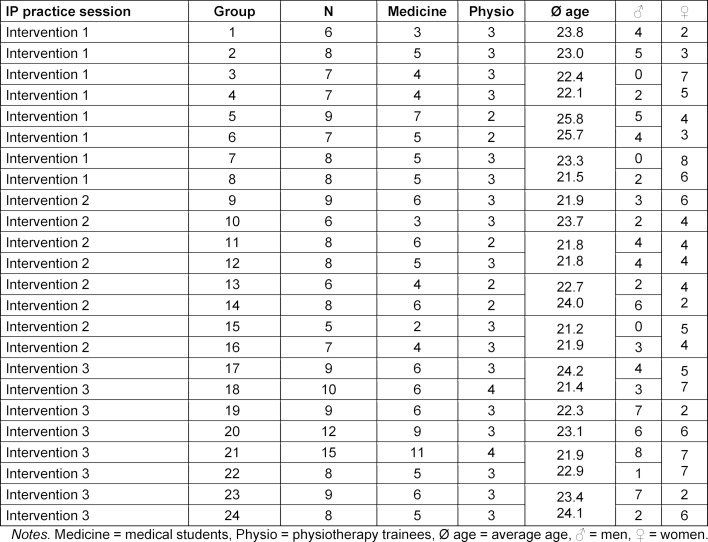
Composition of the IP groups

**Table 3:**
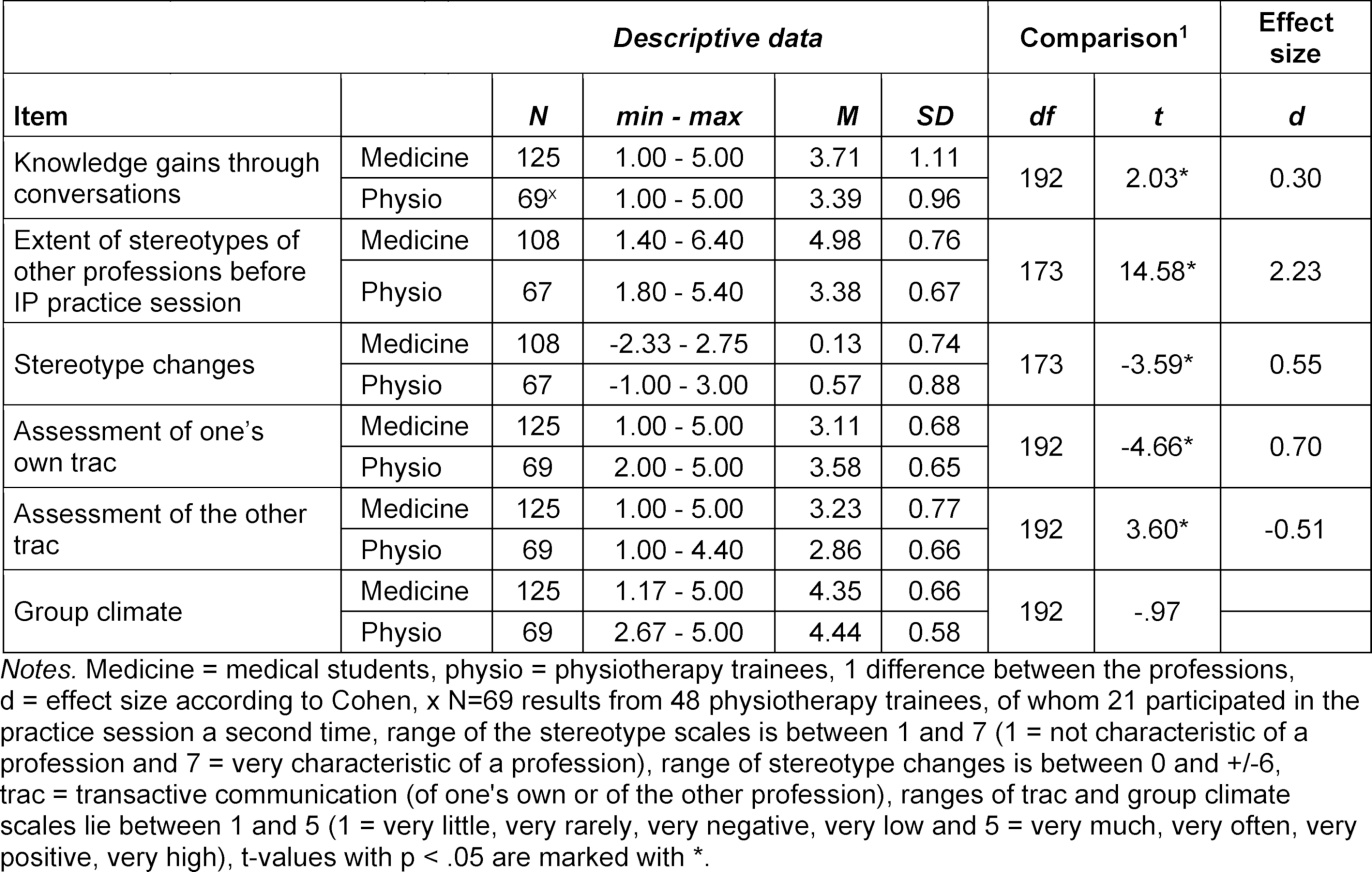
Descriptive data for the IP group work variables with comparison of professions
